# Correction: Pregnant women with mild COVID-19 followed in community setting by telemedicine, and factors associated with unfavorable outcome

**DOI:** 10.1371/journal.pone.0318443

**Published:** 2025-01-24

**Authors:** Aurélien Dinh, Florian Drouet, Agnes Dechartres, Youri Yordanov, Clara Duran, Nicolas Schmidt, Amélie Banzet, Marie-Hermine Perrier, Nathalie Mosquet, François-Xavier Lescure, Patrick Jourdain, Jacky Nizard, Xavier Masingue, on behalf of the AP-HP/Universities/Inserm COVID-19 research collaboration

There is an error in the author affiliation for Agnes Dechartres. The correct affiliation is as follows: Sorbonne Université, INSERM, Institut Pierre Louis d’Epidémiologie et de Santé Publique, UMR-S 1136, AP-HP, Hôpital Pitié Salpêtrière, Département de Santé Publique, Centre de Pharmacoépidémiologie de l’AP-HP (Cephepi), Paris, France.
